# Diabetes and Technology in Romania: A Patient’s Perspective

**DOI:** 10.7759/cureus.68768

**Published:** 2024-09-06

**Authors:** Andrada Raluca Pop, Béla Kovács, Boglárka Kovács-Deák, Cristina Filip, Gabriela Roman

**Affiliations:** 1 Department 5 – Medical Specialties, Diabetes and Nutritional Diseases, Faculty of Medicine, "Iuliu Hațieganu" University of Medicine and Pharmacy, Cluj-Napoca, ROU; 2 F1/Biochemistry and Chemistry of Environmental Factors, Faculty of Pharmacy, George Emil Palade University of Medicine, Pharmacy, Science, and Technology of Târgu Mureș, Târgu Mureș, ROU; 3 The Doctoral School of Medicine and Pharmacy, Institution Organizing University Doctoral Studies, George Emil Palade University of Medicine, Pharmacy, Science, and Technology of Târgu Mureș, Târgu Mureș, ROU; 4 F1/Biochemistry and Chemistry of Environmental Factors, Faculty of Medicine in English, George Emil Palade University of Medicine, Pharmacy, Science, and Technology of Târgu Mureș, Târgu Mureș, ROU

**Keywords:** diabetes care, glucose monitoring systems, insulin pumps, medical devices, questionnaires

## Abstract

Professional medical care in the case of diabetes is of utmost importance to ensure patient health, compliance, and comfort. In the past decades, the emergence of healthcare medical devices has also brought important advancements in diabetology. However, this also raised new provocations for patients and healthcare professionals as well, regarding the acceptance, use, and contentment of sensors and pumps in the everyday lives of diabetic patients. The present study aimed to bring more evidence into the possibilities and pitfalls of these medical devices by interrogating 185 diabetic patients through online questionnaires from Romania. The results revealed that the medical devices can complement traditional medical care, and pre-, post-prandial, and nighttime glycemia can be more precisely achieved. Patients have also reported that the sensors and pumps can augment their daily decision-making about glycemic control and ease their daily routine. Contrariwise, the use of these medical devices is related to comfortlessness during sleeping and physical activity. Researchers acknowledge that patients’ information, education, and diabetes management, through the opinion of the patients, can augment patient-focused decision-making in the daycare of diabetic patients.

## Introduction

Diabetes is a significant public health issue, with its prevalence and incidence steadily increasing, particularly in developing countries. The International Federation Atlas estimates that 1,211,900 children and adolescents under the age of 20 have type 1 diabetes [1]. The Diabetes Control and Complications Trial and its follow-up study, the Epidemiology of Diabetes Interventions and Complications Study, have confirmed an improvement in support of medical professionals, leading to a decrease in the occurrence of complications and a delay in progression where it already exists in type 1 diabetes, in adolescents and adults [2]. The treatment of type 1 diabetes aims to keep and maintain blood glucose levels as close to the reference range in people without diabetes as possible [3].

Diabetes technology encompasses hardware, devices, and software designed to help individuals with diabetes enhance glycemic control and improve quality of life [4]. Historically, this technology involved insulin delivery through syringes, pens, and pumps, as well as glycemic monitoring using glucometers and/or continuous glucose monitoring (CGM) systems [4-6].

Insulin therapy employing an insulin pump was introduced at the end of the 1970s, and in Romania, the use of insulin pumps for patients with type 1 diabetes was introduced in 2002 in Cluj-Napoca [7]. The quality of life of people with type 1 diabetes who use insulin pumps has improved considerably by having lower levels of glycated hemoglobin and by experiencing less frequent and less severe hypoglycemia [8,9].

Along with insulin therapy, glycemic self-monitoring plays an important role in type 1 diabetes. In recent years, CGM systems have become the standard of care in type 1 diabetes in several countries, especially among children, adolescents, and young adults [10], being successfully used in insulin-treated type 2 diabetes [11]. Among the benefits of using CGM systems are a lower level of glycated hemoglobin, fewer frequent hypoglycemia episodes, as well as an increase in the span within the target [9]. The most used glucose monitoring systems are real-time CGMs, some of which are called adjunctive, i.e., the user has to measure the blood glucose level with a glucometer to decide on the treatment, such as insulin dose or treatment of hypoglycemia, while the non-adjunctive types do not require calibration [12-14].

The use of CGMs led to the discovery of new indicators of glycemic control, including the number of days of wear, the percentage of time in which the CGM is active, the average blood glucose level, estimated glycated hemoglobin [15], and glycemic variability [16].

After the glucose monitoring system was introduced in the pump technology, a wide spectrum of systems ensued, like sensor-augmented pump systems and automated insulin delivery (AID) systems. The final goal is to obtain a fully closed-loop system [8].

Technology in medicine progresses at a very fast pace, which means there are constant improvements aimed at ensuring control, safety, and comfort for people with type 1 diabetes [17]. Information on the utilization of diabetes healthcare devices in Romania is limited, as outlined in a factsheet from the International Diabetes Federation. Only recently have there been efforts to enhance diabetes management on a broader scale, albeit somewhat hindered by the COVID-19 pandemic [18]. In a study published by Urzeală et al., data from 100 pediatric patients aged seven to 17 showed that 40% of the children used insulin pumps, while 60% managed their diabetes with multiple daily injections [19]. Therefore, we would like to see the level of information patients in Romania have regarding medical technology in type 1 diabetes.

Given the limited information on diabetes healthcare in Romania, our goal was to use an online questionnaire to assess diabetic patients’ awareness of current medical devices, their applications, and ease of use. The survey also aimed to gather insights into patients' attitudes toward different glucose monitoring systems and to understand their needs and preferences regarding the use of these medical devices.

## Materials and methods

Anonymous research was conducted in 2023 to analyze the level of knowledge and use of technology in type 1 diabetes among people with type 1 diabetes in Romania. The research has been approved by the Ethics Committee of “Iuliu Hațieganu” University of Medicine and Pharmacy, Cluj-Napoca, Romania (approval number DEP139/04.05.2022).

Data collection was done through an online questionnaire, distributed in Romania. The questionnaire included 56 questions and was structured in four parts. The first part of the questionnaire was general, with questions about age, sex, environment, age of onset of diabetes, and general knowledge about modern technology used in diabetes. The second part of the questionnaire was addressed to patients who own and use modern devices (conventional insulin pumps, sensor-coupled insulin pumps, and CGM systems). The third part of the questionnaire referred to those who do not currently own a modern device but will soon use it, and the last part was addressed to people who do not own but do not want to use a modern device for glucose monitoring or insulin administration in the near future.

The questionnaire was addressed to people with type 1 diabetes and the parents/legal guardians of the patients under the age of 18, respectively; it was sent anonymously, and the Google Forms platform was used for data collection. The questionnaire is presented in Appendix A.

Data evaluation was conducted using built-in functions in Microsoft Excel (Microsoft Corporation, Redmond, WA, USA). Statistical analysis was performed using GraphPad v3.06 (GraphPad Software Inc., Boston, MA, USA), while multivariate data analysis utilized SIMCA® 18 software (Sartorius Stedim Biotech, Göttingen, Germany). Statistical significance was considered at p < 0.05. Principal component analysis (PCA-X) models were established for pattern recognition and data grouping. Class differences were examined through orthogonal partial least squares - discriminant analysis (OPLS-DA) models, assessing predictive capacity (Q2) and explaining variability (R2X and R2Y). Loading column plots identified variables with discriminatory power, facilitating effective comparison between groups based on the direction and magnitude of loading coefficients.

## Results

The survey was completed by a total of 185 persons, consisting of 137 female and 48 male patients, representing 74% and 26% of all respondents, respectively. Approximately two-thirds of the participants reside in urban areas (69%, n = 127), while the remaining one-third live in rural environments (31%, n = 58). The age distribution of the respondents is detailed in Table 1.

**Table 1:** Age distribution of survey respondents

Age category	Number of respondents
Parent of a minor	62
18-24 years	23
25-34 years	48
35-44 years	40
45 years or above	12

The results for question Q6 indicated no statistically significant differences between the responses of female and male patients. Specifically, 73.72% of female patients (n = 101 of 137) and 72.92% of male patients (n = 35 of 48) reported being fully aware of the medical devices used for diabetes management in Romania. Additionally, about one-quarter of respondents in both groups (36 female and 12 male participants) had some information on the topic. Only one respondent indicated having no knowledge of these healthcare devices. No gender-based statistical differences were observed; however, differences were noted based on the area of residence (Table 2).

**Table 2:** Awareness of medical devices according to gender and area of residence Note: The significance is reported for p < 0.05.

Answer	Gender		Habitat	
	Female	Male	Rural	Urban
Yes, a lot (N)	101	35	34	102
Yes, some information (N)	36	12	23	25
No, nothing (N)	0	1	0	1
p	p = 0.920		p = 0.056	

**Figure 1:**
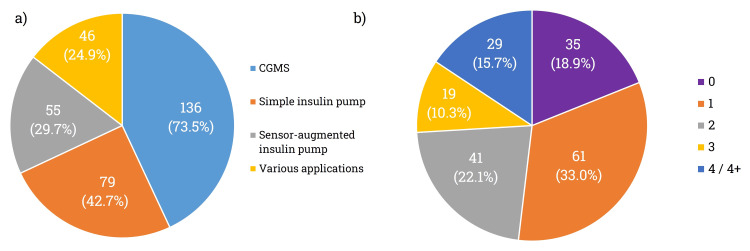
(a) Number of patient’s information about the medical technology used in Romania. (b) Patient awareness of different informational sources

Regarding the responses gathered for question Q7 (Figure 1a), approximately three-quarters (73.5%, n = 136) of the patients were aware of the existence of CGMSs, and nearly half (42.7%, n = 79) were familiar with simple insulin pumps. Unfortunately, only a quarter of respondents were aware of the potential applications of sensor-coupled insulin pumps (29.7%, n = 55) or other advanced applications (24.9%, n = 46). More than half of the respondents had knowledge or heard of one or two possibilities regarding these medical possibilities (Figure 1b). The remaining respondents were almost evenly split between those who had no information about the diabetes-related devices (18.9%, n = 35) and those who were well-informed (15.7%, n = 29).

By analyzing Q7 based on the residence of the respondents, there is a statistically significant difference between the two groups (p = 0.008).

**Figure 2:**
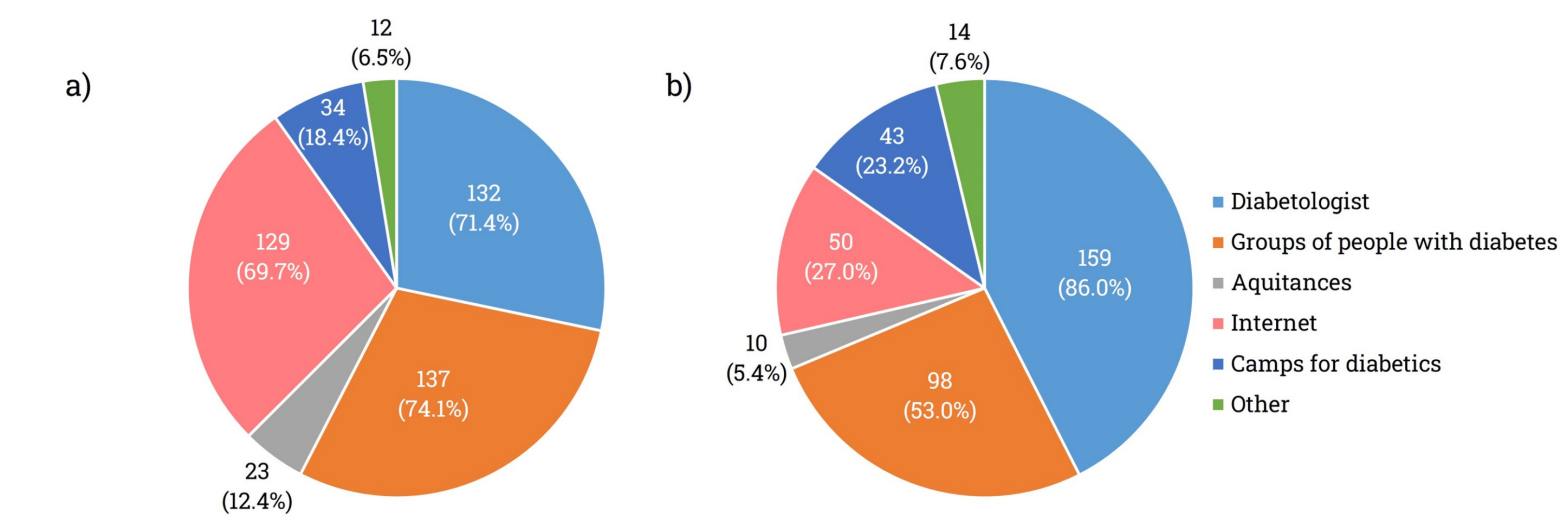
(a) Depiction of the most important information channels used by diabetic patients. (b) Reliability of the provided information according to diabetic patients Values indicate the number of respondents (n).

Regarding the primary information channels (Q8), the majority of respondents identified their diabetologist (71.4%, n = 132), diabetes support groups (74.1%, n = 137), and the internet (69.7%, n = 129) as sources of guidance. Other channels, such as acquaintances, diabetic camps, and miscellaneous sources, were considered less significant among those surveyed (Figure 2a). While 86% (n = 159) of respondents viewed their treating physician as a reliable source of information, over half regarded diabetic support groups as trustworthy. Only about 25% of the participants trusted the internet (n = 50) and diabetic camps (n = 43) as reliable sources, whereas fewer than 10% placed trust in the opinions of friends (n = 10) or other sources (n = 14) (Q9, Figure 2b).

**Figure 3:**
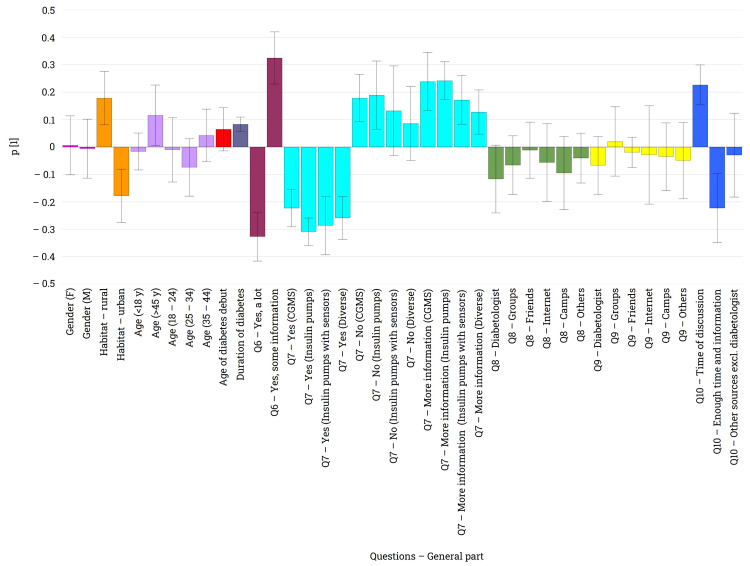
Loading plot illustrating the most important variabilities in the data obtained for Q6-Q10 and the direction of this information The loading bars indicate the direction and magnitude of the correlation between the two variables. The error bars indicate if the selected parameters are correlated in a statistically significant manner.

The exploratory data analysis has validated the statistical findings presented and unveiled significant insights into the survey (Figure 3). As already disclosed, concerning all information from Q6 to Q10, no gender-dependent differences were identified, indicating that the level of knowledge or acquisition of information about glucose monitoring systems is equivalent among female and male patients.

Noticeable disparities emerged between urban and rural patients, with urban patients exhibiting a broader knowledge of these devices compared to rural patients, who possessed only partial information. This contrast was particularly striking when examining responses to Q7, where urban patients provided significantly more positive answers regarding their knowledge of these systems (Q7 - Y bars vs. Q7 - N bars). Presumably, the lack of knowledge among rural patients motivates them to seek further information, as indicated by their interest in learning more about these devices (Q7 - Info bars). Regarding the informational channels and the trustworthiness of these (Q8 and Q9), there is no sex, age, habitat, or any other differences between the analyzed groups. Given the present circumstances, individuals with limited familiarity with these medical devices (Q6 - R2) and those who do not use any of them (Q7 - N) expressed a need for further discussion and information from their diabetologist regarding the latest diabetes devices. Conversely, those who answered affirmatively to both questions feel adequately informed through discussions with their physician (Q10).

Part A: For those who already use a modern device

**Figure 4:**
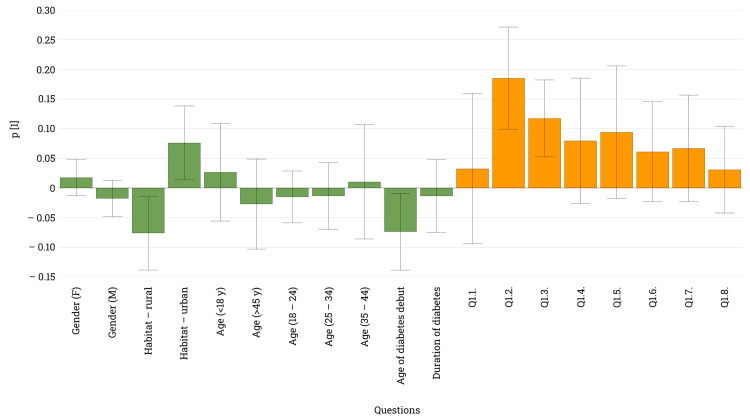
Loading plot illustrating differences between gender, age, habitat, and diabetes antecedents in the context of utilizing modern medical devices

Analyzing the utilization of modern devices for monitoring blood glucose levels (Q1), it was revealed that the vast majority of respondents (93.5%, n = 173) are currently using one of the listed options, with approximately half of them (49.1%, n = 85) using more than two different devices for this purpose. Among these, CGMs were the most commonly used, employed by 84.4% (n = 146) of all patients. Insulin pumps, as well as sensor-augmented insulin pumps, were utilized by approximately 40% (n = 74) of the patients. Other options, such as glucose management software or alternative applications, were utilized by less than a quarter of participants. The loading plots generated (Figure 4) indicated that modern glucose management technologies were generally used by patients residing in urban areas, and this is more pronounced when considering insulin pumps with or without sensors (bars Q1.2 and Q1.3). In terms of gender or age, no differences were identified. Another interesting observation pertains to the age at which diabetes first manifests and its correlation with the utilization of insulin pumps. Present regulations often establish more permissive guidelines for younger patients, particularly children.

More than 90% (n = 168) of patients perceived the use of these medical devices as beneficial for managing diabetes (Q2 and Q6), and they currently utilize them (Q3), while only a small minority indicated occasional or perceived no advantages associated with their use. Among the patients who are currently using some type of device, the majority were using sensors from Medtronic Plc., followed by Dexcom and Free Style Libre.

The duration of device utilization among respondents varied considerably, ranging from patients who have recently started using these devices (around three months) to others who have been using them for 10 years. The average duration within the studied sample was 2.9 years, with a median value of two years (Q5).

**Figure 5:**
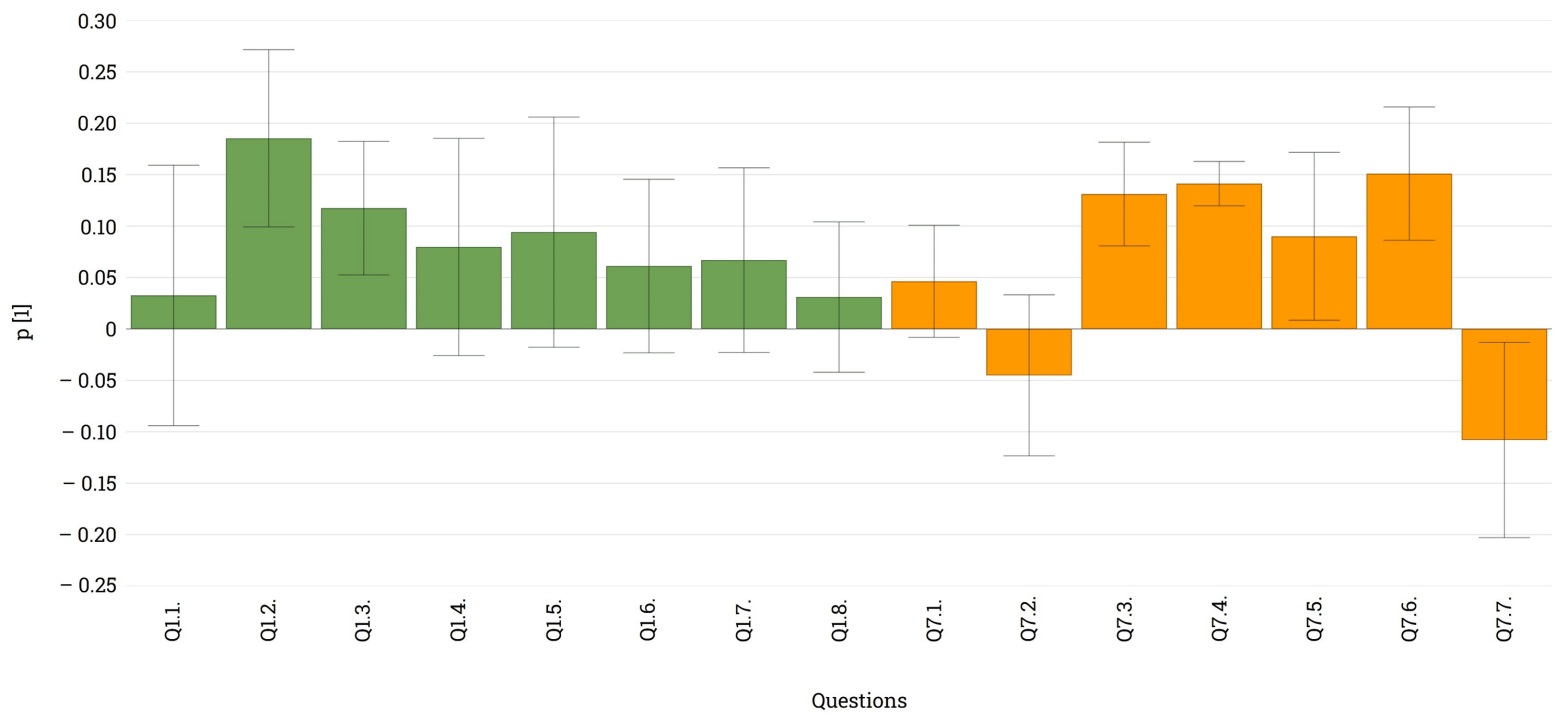
Loading plot revealing the relationship between different types of medical devices used and advantages associated with them

Overall, patients using modern glucose monitoring systems generally reported beneficial improvements in glycemic control (Q7, Figure 5). Specifically, those using insulin pumps with or without sensors (Q1.2 and Q1.3) observed notable enhancements in their glycemic profiles (Q7.3), including reduced blood sugar level fluctuations (Q7.4) and fewer hypoglycemic events (Q7.5). These factors combined may contribute to improved glycated hemoglobin levels (Q7.6), which are critical for diabetic patients. All respondents using some type of device experienced fewer hyperglycemic states (Q7.1) and reported modifications in their glycemic profiles (Q7.7). The reliability of these devices was underscored in the responses to Q8, where only 25% (n = 40) reported encountering issues, and another 25% (n = 45) experienced problems only occasionally. Notably, half of the users (n = 83) have never experienced any problems or difficulties. For those who did encounter issues, the main concerns were technical, such as sensor errors or inaccurate readings, app malfunctions, or significant discrepancies in measurements compared to traditional glucometers, particularly during calibration (Q9).

**Figure 6:**
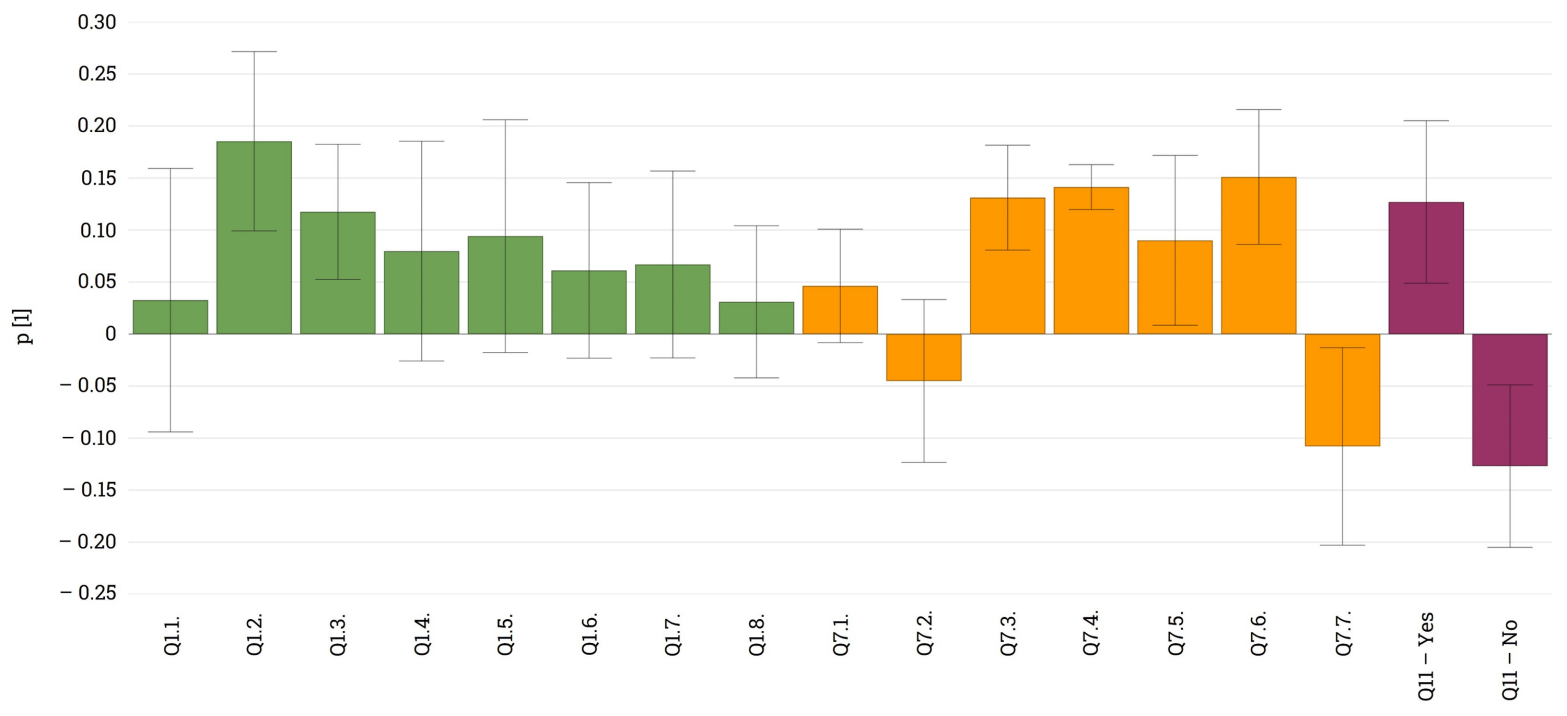
Loading plot revealing the relationship between the advantages of different types of medical devices and improved diabetic control

The vast majority (91%, n = 153) of patients have declared that medical devices give them security in the decision-making process (Q10), and if we analyze Q11, it is noteworthy that the combined effect of using medical devices and taking real-time decisions contributes significantly to a reduction in hypoglycemic events, thus reducing the abundance of this life-threatening state (Figure 6). Patients also acknowledged that since using the sensor, they have learned to manage hypoglycemia more effectively (Q13), giving them better daily flexibility (Q14).

Concerning the wearing of medical devices (Q12), only four individuals reported finding it burdensome or uncomfortable, while 57.4% (n = 97) indicated no issues with wearing these devices, and 40% (n = 68) admitted to occasionally experiencing discomfort. This is also confirmed by the answers given to Q15, Q16, and Q18, suggesting that the patients were relatively comfortable with wearing the sensors while sleeping or playing sports (Table 3).

**Table 3:** Distribution of answers given to Q8-Q18

Answers	Questions									
	Q8	Q10	Q11	Q12	Q13	Q14	Q15	Q16	Q17	Q18
Yes, n and %	40 (23.8%)	153 (90.5%)	149 (88.2%)	4 (2.4%)	162 (96.4%)	166 (98.2%)	10 (5.9%)	8 (4.8%)	57 (33.9%)	1 (0.6%)
No, n and %	83 (49.4%)	1 (0.6%)	20 (11.8%)	97 (57.4%)	6 (3.6%)	3 (1.8%)	159 (94.1%)	160 (95.2%)	111 (66.1%)	142 (84.5%)
Sometimes, n and %	45 (26.8%)	15 (8.9%)	-	68 (40.2%)	-	-	-	-	-	25 (14.9%)
SUM, n	168	169	169	169	168	169	169	168	168	168

When patients were asked whether using the sensor significantly limits their social life, 72% (n = 122) responded negatively, while 18.5% (n = 31) reported that it can occasionally be uncomfortable (Q19). The remaining respondents acknowledged that alarms from the sensor can be disruptive, but they sometimes silence them to avoid interruptions.

**Figure 7:**
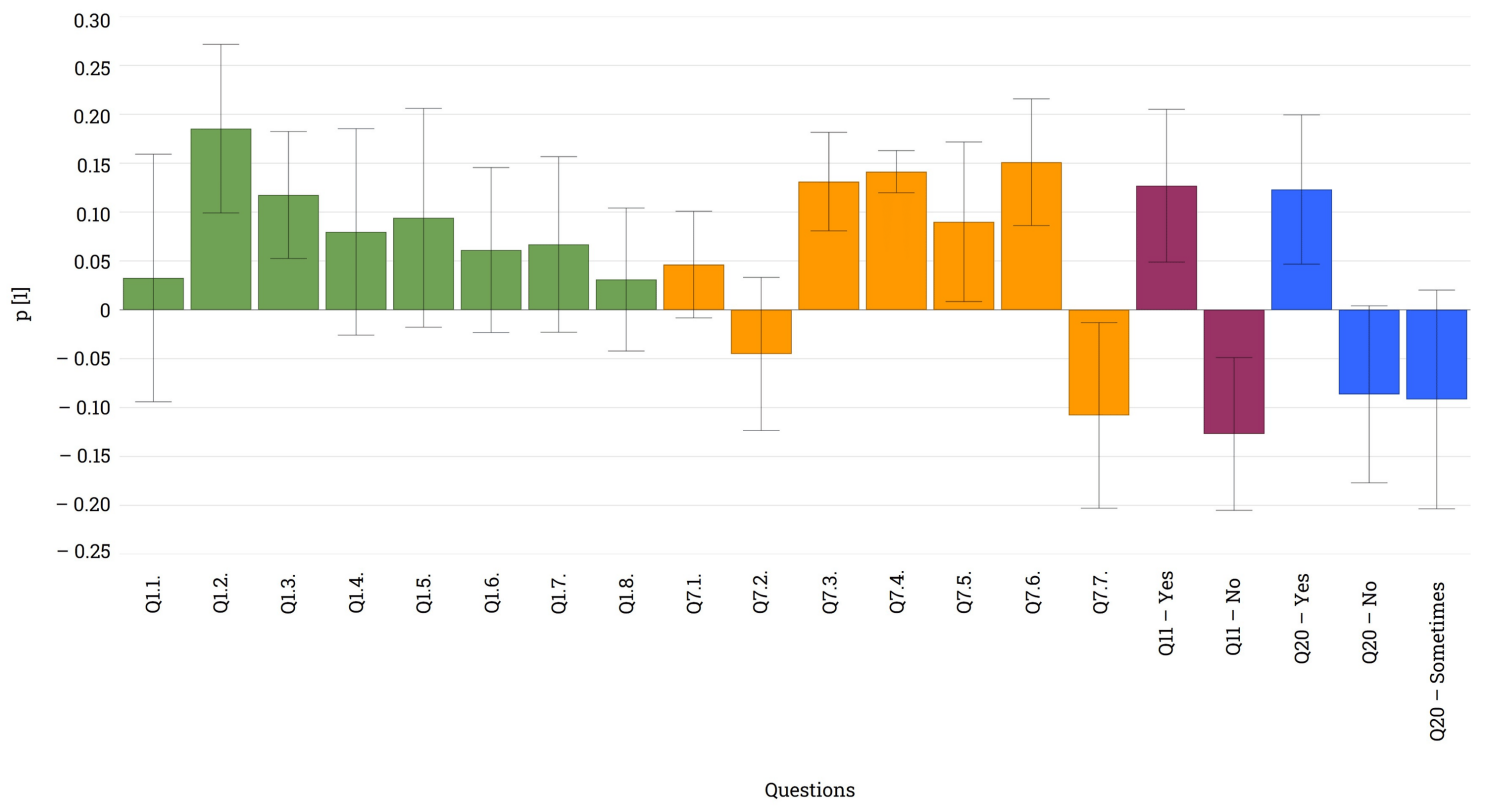
Loading plot revealing relationship between the advantages of different types of medical devices and improved night insulin control

Another significant advantage of these medical devices was highlighted by the responses to the upcoming questions (Figure 7). Not only can patients manage hypoglycemic episodes more effectively, as mentioned earlier, but the sensors can also assist in adjusting night insulin (Q20) and pre-prandial insulin doses (Q21). In the former case, this led to a significantly higher number of positive responses. Moreover, data registration and historical data analysis present another significant advantage of these systems. Sixty-four percent of patients (n = 107) download data from the device, with half of them sending it directly to their diabetologist (n = 58), while 16% (n = 27) analyze it together with the physician during medical check-ups. Only 12.5% (n = 21) of the patients acknowledged having no knowledge about the methods for downloading data (Q22). These remarkable advantages might represent the overwhelming, almost 100% (n = 168) negative answer to Q23, which inquires about the possibility of renouncing the use of such medical devices.

Examining patients’ requests for further enhancement of these medical devices highlighted several additional needs (Q24). These include improved measurement accuracy with fewer calibration periods, extended sensor life span, enhanced accessibility to the devices through improved healthcare systems or favorable pricing, and finally, consideration for the aesthetics and dimensions of the sensors and pumps.

**Figure 8:**
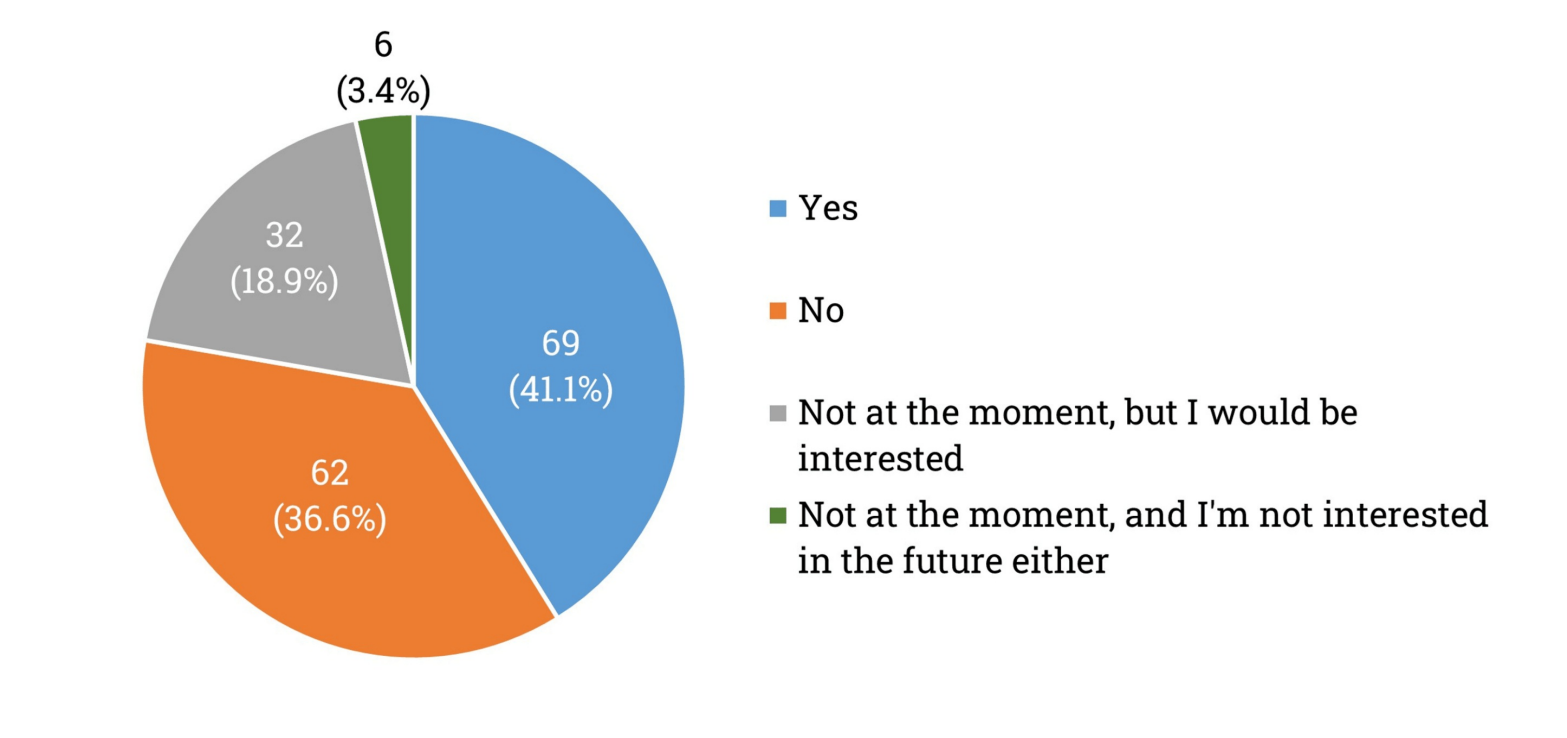
Distribution of insulin pump usage among the surveyed patients (%)

The survey also found that 41.1% (n = 69) of patients were using insulin pumps, while 36.6% (n = 62) were not using this technology. Additionally, 18.9% (n = 32) of respondents who were not using pumps showed interest in utilizing them, while 3.4% (n = 6) were not interested in using insulin pumps now or in the future (Q25, Figure 8).

As in the case of sensors, the average use of these devices was 3.4 years (0.25-15 years), with a median value of two years (Q26). Among the patients who used insulin pumps (72 persons), three-quarters used pumps without sensors, and only a quarter had sensor-augmented insulin pumps (Q27). As in the case of sensors, insulin pumps were also considered to ease the control of blood glucose levels and implicitly the diabetes itself (Q28), as 70% of the respondents have witnessed a better, more flat glycemic profile (Q29).

**Figure 9:**
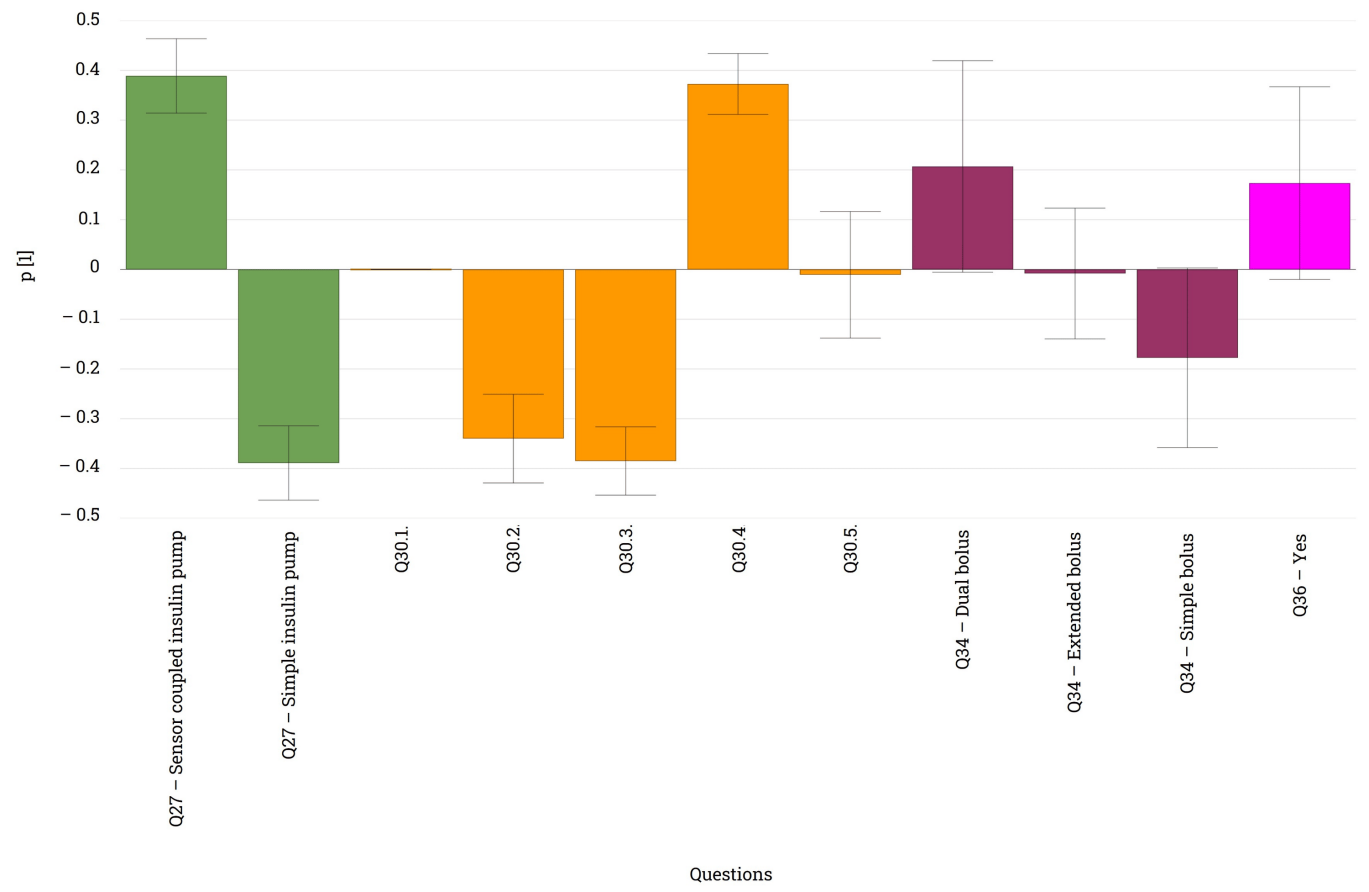
Loading plot illustrating the association between the type of insulin pumps used and the advantages associated with these devices

Among the advantages associated with insulin pump use, the most chosen were the reduced number of injections and better glycemic control. Additionally, the ability to administer different types of boluses and set temporary rates (Q30) was also considered beneficial. This sentiment was echoed in the responses to Q33, with 91.6% (n = 66) of respondents believing that the ability to administer different types of boluses facilitated easier adjustment and correction of various glycemic states. Differences in perceived advantages were observed between respondents using pumps with or without sensors. Those using sensor-augmented insulin pumps emphasized the advantage of administering different types of boluses, while patients using pumps without sensors highlighted easier glycemic control and fewer injections (Figure 9). Convenience and temporary rates were not distinctive for either group. Regarding bolus types, patients using pumps without sensors primarily administered simple boluses, whereas those with sensors preferred dual boluses. Only 10% (n = 8) of patients used extended boluses, which were not characteristic of either group (Q34). A temporary basal rate was applied by approximately 75% (n = 53) of the respondents, who also recognized it positive use during physical activities or on sick or stressful days (Q35, Q36).

The convenience of using insulin pumps is reflected by the answers given to Q37. Over 50% (n = 37) of the patients who use insulin pumps do not feel uncomfortable using them, and 75% (n = 54) consider it a normality in terms of medical care. On the other pole, only a minor fraction of respondents (4%, n = 3) are feeling embarrassed in front of other people. Comfortlessness is mainly reported during sleeping and physical activities, when 16% (n = 12) of the persons with these medical devices have reported this issue. In terms of physical activity, insulin pumps are perceived as more advantageous compared to traditional pens, as indicated by 77% (n = 56) of respondents (Q31). Two-thirds of the patients reported (n = 55) that wearing insulin pumps does not impede their participation in sports activities, while one-third (n = 25) mentioned that insulin pumps sometimes limit their physical activity (Q32).

Patients who used medical devices for diabetes management would unanimously recommend their use to other patients (Q38), and more than 90% considered that a regional center for diabetes care would be an outstanding opportunity for better management (Q40), although 9 out of 10 patients acknowledged that they have sufficient time for discussion with their physician for the management of diabetes (Q39).

Part B: For those who are not yet using modern devices but will follow shortly

Seventy-seven individuals have expressed interest in potentially becoming future users of these medical devices. Among them, 42 have already sought information about the functionality of these devices, while 34 are awaiting medical instructions from their physician. One person had no interest in having further information on these devices (Q1).

The main expectations regarding sensors (Q2) are primarily centered on achieving improved insulin dose adjustment and preventing hypoglycemic episodes (Q2.2 and Q2.3, 71% - n = 54 and 64% - n = 49, respectively), as indicated by approximately two-thirds of the respondents. This aligns well with the responses from Part A, Q11, where patients currently utilizing these devices acknowledge their benefits in managing hypoglycemic events. Half of the patients have noted a hope for better glycemic control (Q2.1, 53% - n = 40) and greater flexibility in daily activities (Q2.5, 51% - n = 39). Finally, approximately 30% of patients have highlighted the advantage of requiring fewer stings (Q2.4, 32% - n = 24) when using glucose monitoring systems.

Patients’ expectations regarding insulin pump usage primarily revolve around insulin dosage (Q3.2, 51% - n = 39), preventing hypo- (Q3.3, 49% - n = 37) or hyperglycemic (Q3.4, 47% - n = 36) events, and the reduction in the number of injections (Q3.5, 47% - n = 36). Approximately 50% of patients have emphasized these aspects as the main anticipated advantages of wearing insulin pumps. As seen in the case of sensors, better glycemic management (Q3.1, 36% - n = 27) together with improved daily flexibility (Q3.6, 37% - n = 28) is of great importance for more than one-third of the respondents. Approximately one-fourth of the respondents have highlighted that reducing the focus on diabetes treatment (Q3.7, 22% - n = 17) and having less liability (Q3.8, 29% - n = 22) in insulin dosage calculation would be another significant advantage of using such medical devices.

Part C: For those who do not want to use any of the modern medical devices

Part C of the questionnaire was completed by 41 of the patients, among whom 22 persons have used in the past a CGM system, whereas 19 persons have never used any of these medical devices (Q1).

Despite the number of respondents to this part of the survey, a relatively small percentage of respondents have managed to express motives not to use sensors or insulin pumps. Six patients provided alternative explanations for abstaining from using these types of medical devices. Three respondents indicated the adequacy of traditional glucometers; two individuals stated they did not perceive a need for such devices; and one person mentioned that it would only complicate their daily routine. In addition to other reasons, some patients cited familiarity with pens and a desire to avoid dependency on a device as obstacles to using insulin pumps. Additionally, two individuals expressed that their lives are easier without pumps, citing potential stress and complications or a lack of confidence in using advanced technologies from a technical standpoint.

## Discussion

The present article aimed to bring insights into patients’ attitudes toward diabetes care in terms of medical devices such as sensors and pumps. Our survey revealed that the accumulated knowledge about the different medical devices varies mainly on the residence of the respondents, as urban residing patients are more aware of the possibilities compared to their rural counterparts. Concerning gender, no differences were observable between female and male patients. A similar, questionnaire-based survey published by Almousa et al. disclosed that the level of knowledge related to diabetes healthcare is significantly correlated with different socioeconomic statuses of the respondents, e.g., age, level of education, accommodation, and monthly earnings. Their conclusion has also underpinned the importance of healthcare education among patients to achieve the desired outcomes regarding diabetes and lifestyle management [20]. Undoubtedly, patient education is of utmost importance when it comes to the acceptance and application of medical devices used in diabetes care. This is also evidenced by our study that rural residing patients have less knowledge about the applicability of CGMSs, simple or sensor-augmented insulin pumps, or other, various applications, whereas urban residing patients have more knowledge about these possibilities. Moreover, rural patients have expressed their need to prolong the time of discussion with their current physician. Inequities between rural and urban patients in terms of diabetes care were also shown by Flood et al. by analyzing diabetes care in 42 low- and middle-income countries. According to their findings in the case of urban patients, a greater number can achieve performance measures of diabetes care in terms of diagnosis, treatment, and control. Their findings also bring attention to the importance of initiating programs and policies to reach the rural population and tarnish differences between residential statuses [21].

Patients who are currently using modern medical devices in diabetes care often report an improvement in glycemic control associated with reduced hyperglycemic states and more optimal glycated hemoglobin levels [22]. Our findings are aligned with literature data, as the interrogated patients have reported beneficial aspects while using glucose monitoring sensors and insulin pumps. In an observational, retrospective study of online survey data obtained from a cohort of 2044 adults with type 1 diabetes, the use of more advanced technologies was associated with a numerically greater proportion of respondents achieving glycemic targets, although despite the use of even the most advanced systems, specifically AID systems, 16.6% of participants reported severe hypoglycemic events in the previous year [3]. Furthermore, a randomized trial published by Laffel et al. demonstrated a small but significant improvement in glycemic metrics when adolescent and young adult participants with type 1 diabetes used CGM compared with self-monitoring of blood glucose [23]. Other retrospective studies focusing on the advantages of CGMs concerning self-monitoring of blood glucose have highlighted the advantages of CGMs in terms of HbA1c changes. The adoption of CGMs not only favored patient decision-making but also contributed to a significant improvement in glycemic control - reduction of HbA1c and average blood glucose levels. Furthermore, the hypoglycemic events have declined, and patients have encountered more time within the target blood sugar range [22]. Besides the improvement of the glycemic profile, our study has also highlighted that patients who are using modern medical devices are more definite in decision-making regarding the regulation of blood glucose levels and adjusting insulin doses. This is also confirmed by the observation that patients are more likely to have better control over the night and postprandial glucose levels, and access to historical data can augment the decisions taken by the patient. Similarly, a large national survey of adults with type 1 diabetes who have used or are using advanced technology to manage their glucose levels indicated that these devices are perceived as having an important role in maintaining healthy glucose levels, reducing restrictions on the activities of daily living, and enhancing emotional well-being and quality of life [24].

Analytical studies have also shown that real-time CGMs can augment not only the daily routine of diabetes patients but also contribute to reducing anxiety and pain sensations and ensure uninterrupted sleep compared to self-monitoring blood glucose patients by magnitudes of impact [25]. Obediently, our data has also shown that convenience in terms of easing the daily routine and quality of life of patients using glucose monitoring systems is one of the main reasons for which renouncement to these medical devices is not considered. Nevertheless, patients who only intend to use diabetes care medical devices defined flexibility and reduced number of hypo- and hyperglycemic events as primary expectations vis these healthcare gadgets. Those who are not planning to use sensors or pumps reported the complicatedness and stresses associated with the wearing of these medical devices.

Although this research aimed to assess the level of knowledge as broadly as possible in Romania, generalizing the findings to the entire population may be limited. The subjective nature of responses may not adequately capture variability within different subgroups. Additionally, response and recall biases could affect the accuracy of the data, as participants’ ability to interpret questions may be influenced by their level of knowledge or medical history, potentially leading to overreporting of certain information or behaviors. It is also important to acknowledge that the cross-sectional study design only identifies associations between variables and does not establish causality. Despite these limitations, the study offers valuable insights into the knowledge and practices of diabetes patients in Romania and can serve as a foundation for future research in public healthcare and understanding patients’ needs in diabetes management.

## Conclusions

The technology used in diabetes improves the lives of patients to a considerable extent, the most widely used devices being CGM systems, followed by conventional insulin pumps or insulin pumps coupled with CGM systems. These technologies offer numerous benefits, including lower glycated hemoglobin levels, reduced frequency and severity of hypoglycemia episodes, greater daily flexibility, and better maintenance of glycemic profiles within target ranges both day and night. Regarding the use of insulin pumps, patients have noticed benefits in the glycemic profile, its improvement, the reduction of the number of injections, and also the use of different types of boluses and the temporary basal rate; 91% of all respondents reported that the use of medical devices gives them security in daily decision-making. More than 50% of patients expressed satisfaction with these devices, noting that they provide a sense of security and do not cause discomfort during sleep or physical activities, and more than 75% of the respondents consider the use of the medical devices as a normality in terms of medical care. However, they hope future devices will be more accurate in transmitting glycemic data, require fewer calibrations, have longer sensor life, be more affordable or accessible through the national healthcare system, and feature more discreet designs.

On the other hand, patients who do not yet use sensors but plan to do so anticipate that these will contribute to the reduction and prevention of hypoglycemia episodes, improvement in the adjustment of insulin doses, greater flexibility in daily activities, and also fewer stings. Patients’ expectations regarding insulin pumps revolve primarily around the dose of insulin, the prevention of episodes of hypoglycemia and hyperglycemia, and the reduction of the number of injections. Less than 10% of the respondents expressed reluctance to use the currently available devices, preferring to monitor their blood sugar with traditional glucometers and administer insulin using pens. These patients feel that these devices might complicate their daily routines, increase stress, create dependency, and are not fully confident in the reliability of the technology.

## Appendices

Appendix A

**Table 4:** Level of knowledge and use of technology in the management of type 1 diabetes mellitus in Romania

Gender
Male
Female
Residence
Urban
Rural
Age
18-24 years
25-34 years
35-44 years
> 45 years
Parent of a minor
Age of diabetes debut: ____________ (open question)
Duration of diabetes: _____________ (open question)
Do you have any information about the diabetes medical technology in Romania (blood glucose monitoring sensors, insulin pumps, insulin pumps augmented with continuous glucose monitoring systems, smart pens for insulin administration, various applications, programs)?
Yes, a lot
Some information
No, not at all
Do you think you have enough information about the medical technology used in Romania?
Continuous glucose monitoring systems Simple insulin pumps Sensor augmented insulin pumps Diverse applications Yes ¨ ¨ ¨ ¨ No ¨ ¨ ¨ ¨ Would like more information ¨ ¨ ¨ ¨
Where do you get information on modern technology used in Romania? (multiple answers)
Healthcare professional
Groups of people with diabetes
Friends
Internet
Camps for diabetic patients
Other
Which sources of information do you consider to be more accurate and reliable, and which do you prefer? (multiple answers)
Healthcare professional
Groups of people with diabetes
Friends
Internet
Camps for diabetic patients
Other
Given the current situation, do you feel that you would need more discussion and information from your diabetologist about new diabetes devices?
Yes, I would like more time for discussion and more information
The discussion time and information I have is sufficient
I get more information from other sources than from my diabetes doctor
I prefer to get information from the internet
Part A – for those who already use a modern device
Do you or your child currently use: (multiple answers)
Continuous glucose monitoring systems - sensor
Insulin pumps
Insulin pumps augmented with continuous glucose monitoring systems
Nutritional (phone) apps
Physical activity monitoring apps (pedometers)
Glucose management programs (such as MYSugr)
Insulin dose calculation applications
Other applications
Do you consider that the use of technology will help you to better manage diabetes?
Yes
No
Sometimes
Do you currently use a glucose monitoring system (SENZOR)? If YES, continue with the questions below; If NO, continue from question 25.
Yes
No
Which sensor do you use? (optional answer) ______________ (open question)
How long have you been using the sensor? _______________ (open question)
Do you consider the sensor useful?
Yes
No
Sometimes
Have you noticed an improvement in diabetes control since using the sensor? (multiple answers)
Blood sugars have decreased
Blood sugars have increased
Glycaemic profile is flatter
Blood glucose variations have decreased
Reduced hypoglycaemia
Glycated haemoglobin significantly improved
No change in glycaemic values
Over time, have you encountered any difficulties in using the sensor?
Yes
No
Sometimes
If you encountered difficulties in using the sensor, what were they? (If no, do not answer this question): ___________________________________ (open question)
Since using the sensor, do you feel more confident in making decisions?
Yes
No
Sometimes
Are episodes of hypoglycaemia rarer since using the sensor?
Yes
No
Is the sensor uncomfortable/painful?
Yes
No
Sometimes
Since using the sensor have you learned to treat hypoglycaemia more correctly?
Yes
No
The sensor gives me more freedom/flexibility in my daily life?
Yes
No
Was using the sensor more complicated than you expected?
Yes
No
Does wearing the sensor bother you during sleep?
Yes
No
Do the sensors show more errors than you expected?
Yes
No
Does the use of the sensor greatly limit sports, outdoor activities?
Yes
No
Sometimes
Does the use of the sensor greatly limit your social life (school, group of friends, society, vacations)?
Yes, I embarrass myself in front of others
No, I consider a normal and useful thing
Sometimes, in certain situations but not frequently
Alarms bother me
I usually turn off alarms
Using the sensor helps me to adjust insulin doses during the night, when necessary?
Yes
No
Sometimes
Does the use of the sensor help me in adjusting pre-prandial insulin doses?
Yes
No
Sometimes
Who downloads periodically reports and data from the monitoring system?
The diabetologist in consultation
I personally download to analyse glycaemic values
I personally download and send to the diabetologist
I easily use the download system
Don't know what downloading data involves
Would you give up the sensor?
Yes
No
What would you improve about the sensor system? (open question)
Do you currently use insulin pumps?
Yes
No
Not currently, but I would be interested
Not now, nor am I interested in the future
If yes, what type of pump do you use? ________________ (open question)
If yes, which insulin pump do you use?
Simple insulin pump without sensor connection
Insulin pump with sensor, e.g. Minimed 640G, Minimed 780G
Do you find that using an insulin pump will help control your diabetes, blood sugars are easier to control?
Yes
No
Since using the insulin pump has glycaemic control improved, has the glycaemic profile become flatter?
Yes
No
It remained almost the same
Considerable improvement
The biggest advantage of using insulin pumps? (multiple answers)
More convenient
Easier to control blood sugars
Fewer injections
Different types of boluses
Using the temporary rate
In terms of daily activities and sports, do you consider insulin pumps to be more useful than multiple pen injections?
Yes
No
Sometimes
Does the use of an insulin pump greatly limit sports, outdoor activities?
Yes
No
Sometimes
Does the use of an insulin pump help me to adjust insulin doses and to correct blood glucose by different types of boluses?
Yes
No
Sometimes
What types of boluses do you use most frequently?
Simple bolus
Extended bolus
Dual bolus
Do you use temporary basal rate?
Yes
No
I prefer to correct blood glucose by bolus or pump off
Do you think it will help the temporary basal rate during physical activity or on sick/stressful days?
Yes
No, I do not use the temporary basal rate
Sometimes
Wearing insulin pump ... (multiple answers)
Does not bother me
Bothers me during sleep
Bothers me during the day
Bothers me during sports
Bothers me in society, school, vacations
Embarrass me in front of others
Is a normal and useful thing to do
The use of technology in diabetes helps me, so I would recommend it to other patients?
Yes
No
Compared to the current situation, do you feel that you would need more discussion and information from your diabetologist about insulin pump therapy?
Yes, I would like more time for discussion and more information
The discussion time and information I have is sufficient
I get more information from other sources than from my diabetes doctor
I prefer to get information from the internet
Do you consider it useful to have a Regional Medical Centre specialized in insulin pumps and technology, even if it is further away from home?
Yes
No
Part B – for those who are not yet using modern devices, but will follow shortly
Do you consider that you have enough information about modern devices before installing them?
Yes, I inquired to know as much as possible before installation
No, I prefer to wait for the training given by my diabetologist
Not interested
For SENZOR- What are your expectations from the installation of the continuous glucose monitoring system? (multiple answers)
To regulate my blood sugars
To better adjust insulin doses
Prevent hypoglycaemia
Not need as many injections
Have a more flexible schedule
For INSULIN PUMP- What are your expectations from the insulin pump installation? (multiple answers)
To regulate my blood sugars by itself
To better adjust insulin doses
To prevent hypoglycaemia
Prevent hyperglycaemia
No more injections
Have a more flexible schedule
I don't have to think about my own diabetes treatment
No more calculating insulin doses myself
Part C – for those who do not want to use any of the modern medical devices
Have you ever used one of the devices below and quit?
No, none
Yes, a continuous glucose monitoring system (sensor)
Yes, an insulin pump
What are the reasons why you would not want a continuous glucose monitoring system (sensor)? (multiple answers)
I don't think I need it, it's fine without it
It's enough to measure blood glucose with a glucometer
It's stressful for me to know my blood glucose levels all the time
I don't want people to know I have diabetes
I think it complicates my life
I am not a technical person
Other reasons
What are the reasons why you would not want to use an insulin pump? (multiple answers)
I don't think I need it, it's fine without
I am used to insulin injections
Don't want to depend on a device
It is stressful for me to handle the insulin pump
I don't want people to know I have diabetes
I think it complicates my life
It would interfere with my profession.
I'm not a technical person, I don't understand what to do
Other reasons
